# Cutaneous squamous cell carcinoma in a patient with neurofibromatosis type 1: A case report

**DOI:** 10.3892/ol.2013.1490

**Published:** 2013-07-25

**Authors:** MITSUAKI ISHIDA, HIDETOSHI OKABE

**Affiliations:** Department of Clinical Laboratory Medicine and Division of Diagnostic Pathology, Shiga University of Medical Science, Otsu, Shiga, Japan

**Keywords:** neurofibromatosis type 1, neurofibroma, squamous cell carcinoma, skin

## Abstract

Neurofibromatosis type 1 (NF1) is an autosomal dominant inherited disease that is characterized by the presence of multiple neurofibromas, café-au-lait spots and iris hamartomas. It is well established that the incidence of tumors in patients with NF1 is high compared with the normal population and that the majority of the tumors are non-epithelial neoplasms, including neurofibromas, malignant peripheral nerve sheath tumors, gliomas and leukemia. Studies have suggested that patients with NF1 also have a significantly higher risk of certain types of carcinomas. However, the occurrence of cutaneous squamous cell carcinoma (SCC) in a patient with NF1 is extremely rare. The present study describes the second documented case of a cutaneous SCC adjacent to a neurofibroma of the forehead with histopathological analyses in a patient with NF1. An 80-year-old female with NF1 presented with a rapidly growing skin tumor of the forehead. Histopathological study of the resected forehead tumor demonstrated that there were two tumorous lesions. One was an invasive SCC and the other was a neurofibroma. The lesions were adjacent, but no continuity was present. NF1 is caused by inactivating mutations in the *NF1* gene and loss of heterozygosity of this gene has been reported in neurofibromas, malignant peripheral nerve sheath tumors, gliomas and pheochromocytomas in patients with NF1. However, the genetic mechanism of carcinoma development in patients with NF1 is not well understood. Studies have suggested the role of the *NF1* and/or the *BRCA* gene in the occurrence of breast cancer. Additional studies are required to elucidate these mechanisms.

## Introduction

Neurofibromatosis type 1 (NF1), also referred to as von Recklinghausen disease, is an autosomal dominant inherited disease that affects approximately one in 3,000 individuals (1). The disease is characterized by the presence of multiple neurofibromas, café-au-lait spots, iris hamartomas (Lisch nodules) and axillary and inguinal freckling (1). It is well established that the incidence of tumors in patients with NF1 is high compared with the normal population, which is the main reason for the reduced life span of NF1 patients (2). The majority of tumors arising in NF1 patients are neurofibromas, particularly plexiform neurofibromas, which is a hallmark of this disease. Malignant peripheral nerve sheath tumors also affect these patients. Furthermore, patients with NF1 have a greatly increased risk of developing gliomas, leukemia, particularly juvenile myelomonocytic leukemia, pheochromocytoma and rhabdomyosarcoma (2,3). In addition, certain types of carcinomas, including breast cancer, may also occur more frequently in patients with NF1 (2,4,5). However, the occurrence of cutaneous squamous cell carcinoma (SCC) in patients with NF1 has been rarely documented (6). The present study describes a case of an SCC adjacent to a neurofibroma of the forehead in a patient with NF1. Written informed consent was obtained from the patient.

## Case report

### Patient

An 80-year-old female with NF1 presented with a rapidly growing skin tumor of the forehead. A physical examination revealed numerous cutaneous nodules across the entire body, which were clinically diagnosed as neurofibromas. The forehead skin tumor was well-circumscribed, dome-shaped with a central keratin plug and adjacent to a neurofibroma. The lesion measured 2.5×2.4 cm in diameter. Under the clinical diagnosis of keratoacanthoma, a total resection of the forehead tumor with the adjacent neurofibroma was performed.

### Methods

The formalin-fixed, paraffin-embedded tissue blocks of the resected skin specimen were cut into 3-μm thick sections, deparaffinized and rehydrated. Each section was stained with hematoxylin and eosin and used for immunostaining. Immunohistochemical analyses were performed using an autostainer (XT system BenchMark; Ventana Medical System, Inc., Tucson, AZ, USA) according to the manufacturer’s instructions. A mouse monoclonal antibody against Ki-67 (MM1; Novocastra Laboratories, Ltd., Newcastle upon Tyne, UK) and a rabbit polyclonal antibody against S-100 protein (Nichirei Bioscience, Tokyo, Japan) were used.

### Results

**Figure 1:**
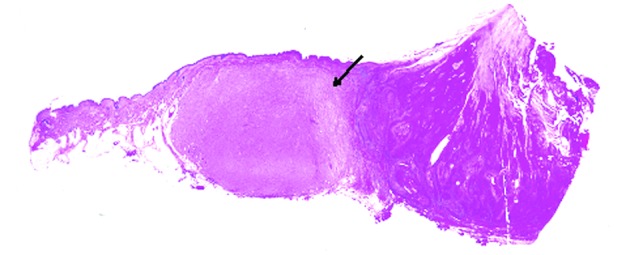
Panoramic view of the forehead tumor. Squamous cell carcinoma (SCC; right) and neurofibroma (left, arrow) are adjacent. However, no continuity is observed (hematoxylin and eosin staining).

Macroscopically, the cut section of the tumor revealed two tumorous lesions. One was a marked hyperkeratotic tumor invading the upper subcutis and the other was a well-circumscribed nodule in the dermis and subcutis (Fig. 1). Although the two lesions were adjacent, no continuity was noted.

**Figure 2:**
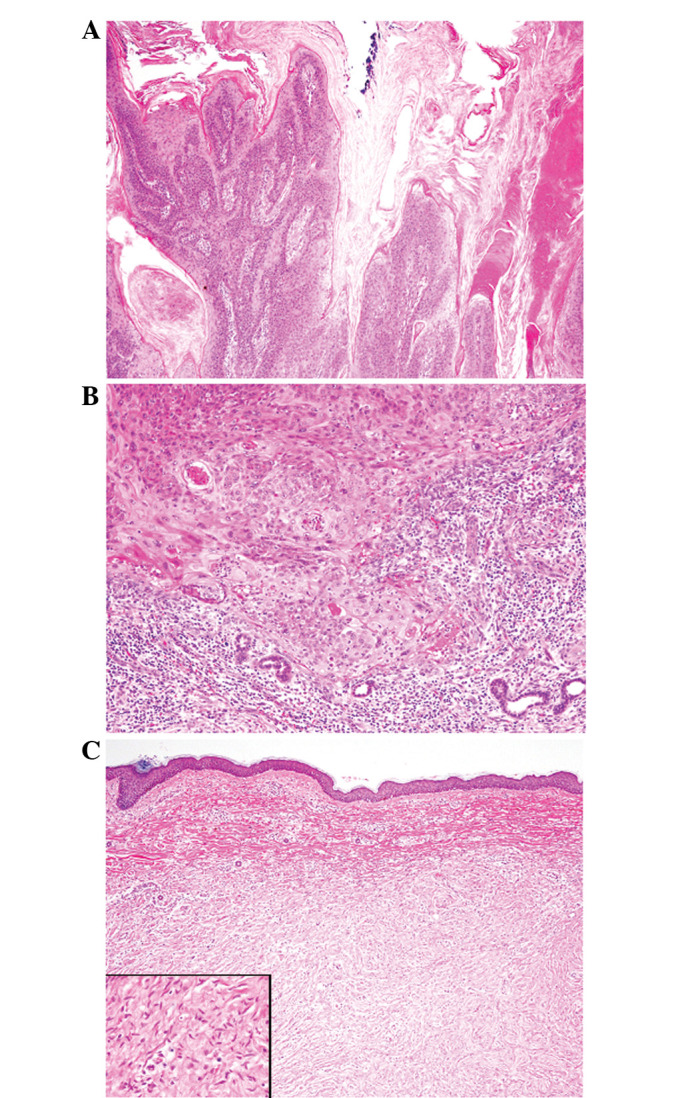
Histopathological observations of the forehead tumor. (A) The SCC is composed of a papillary proliferation of atypical squamous cells with hyperparakeratosis (hematoxylin and eosin staining; magnification, ×40). (B) Tumor cells of the SCC have large nuclei with conspicuous nucleoli and a rich eosinophilic cytoplasm. Peritumoral lymphoplasmacytic infiltration is also observed (hematoxylin and eosin staining; magnification, ×100). (C) The neurofibroma is composed of proliferating spindle cells. These spindle cells have bland cigar-shaped nuclei (inset) [hematoxylin and eosin staining; magnification, ×40 and (inset) ×200]. SCC, squamous cell carcinoma.

Microscopically, the former tumor revealed papillary proliferation of atypical squamous cells with marked hyperparakeratosis (Fig. 2A). These atypical squamous cells contained large nuclei with coarse chromatin, conspicuous nucleoli and a rich eosinophilic cytoplasm (Fig. 2B). Mitotic figures were frequently observed. The tumor had invaded into the upper subcutis and peritumoral lymphoplasmacytic infiltration was also noted (Fig. 2B). These histopathological features were typical for an invasive SCC. The latter component was a neurofibroma, which was composed of proliferating spindle cells containing bland cigar-shaped nuclei with inconspicuous nucleoli and an eosinophilic cytoplasm (Fig. 2C). No mitotic figures were observed. Immunohistochemically, the spindle cells were diffusely positive for S-100 protein and the Ki-67 labeling index was <1%. Therefore, this component was diagnosed as a neurofibroma.

## Discussion

Patients with NF1 are at an increased risk of non-epithelial neoplasms of several types, including neurofibromas, malignant peripheral nerve sheath tumors, gliomas, leukemia, pheochromocytoma and rhabdomyosarcoma (2,3). Furthermore, studies have also suggested an increased risk of certain types of carcinomas in patients with NF1 (2,4,5). Seminog and Goldacre (2) recently analyzed 697 cases of carcinomas in 6,739 patients with NF1. The study revealed that patients with NF1 have a significantly high risk of developing carcinomas of the esophagus, stomach, colon, liver, lung, thyroid, breast and ovary (2). Furthermore, a slightly increased risk of non-melanoma skin cancers was reported, though the histopathological subtypes of the skin cancers were not available (2). However, SCC of the skin accounts for only a small number of malignancies in NF1 patients and to the best of our knowledge, only one case of an invasive SCC of the sole of the foot, with analyses of the histopathological features, has been reported in an NF1 patient (6). The present study is the second documented case of an invasive SCC with histopathological analyses in a patient with NF1.

NF1 is caused by inactivating mutations in the *NF1* gene, which is located on chromosome 17q11.2. This gene has a tumor suppressor function as the gene product of *NF1*, neurofibromin, is a major negative regulator of the RAS/mitogen-activated protein kinase (MAPK) pathway, which transmits mitogenic signals to the nucleus (7). Consistent with Knudson’s two-hit hypothesis, NF1 patients with a heterozygous germline *NF1* mutation develop a somatic mutation in the second wild-type *NF1* allele, resulting in the development of neurofibromas. The loss of heterozygosity of the *NF1* gene is observed in certain types of non-epithelial tumors in patients with NF1. Biallelic *NF1* inactivation is observed in neurofibromas and malignant peripheral nerve sheath tumors (8–10). A high proportion of astrocytomas from patients with NF1 also demonstrate a loss of neurofibromin expression and a loss of heterozygosity of the *NF1* gene (11). Furthermore, the loss of heterozygosity of the *NF1* region has been observed in the majority of pheochromocytoma cases in patients with NF1 (12).

The genetic mechanism of carcinoma development in patients with NF1 is not well understood. However, Güran and Safali (13) reported a case of breast carcinoma in an NF1 patient and loss of heterozyosity of the *NF1* gene in the carcinoma tissue. *BRCA1* or *BRCA2* mutations were not observed in this case. Furthermore, Ceccaroni *et al*(14) reported the cases of five individuals in a family with NF1 who presented with neurofibromas and breast, ovary, peritoneal or rectal carcinomas. The study clearly demonstrated that three individuals shared a common haplotype, including the *NF1* and *BRCA1* loci on chromosome 17 and speculated that the occurrence of NF1 and breast carcinoma in this family was due to the presence of two linked mutations at the *NF1* and *BRCA1* foci (14).

In conclusion, the present study is the second documented case of cutaneous SCC with an analysis of the histopathological features in a patient with NF1. The increased risk of various types of carcinomas, including non-melanoma skin carcinoma, in patients with NF1 is recognized, however, the molecular mechanism of carcinoma development in patients with NF1 is not well understood. Additional studies are required to clarify this mechanism
